# Immune Checkpoint Inhibitor–Induced Myocarditis: A Late Presentation

**DOI:** 10.31486/toj.25.0063

**Published:** 2026

**Authors:** Adrienne Koos, Farnoosh Shariati, Senthil Anand

**Affiliations:** ^1^ Department of Internal Medicine, Ochsner Clinic Foundation, New Orleans, LA; ^2^ Department of Cardiology, Ochsner Clinic Foundation, New Orleans, LA

**Keywords:** *Immune checkpoint inhibitors*, *late onset disorders*, *myocarditis*, *pembrolizumab*, *triple negative breast neoplasms*

## Abstract

**Background:**

Immune checkpoint inhibitors (ICIs) have revolutionized oncology by providing a new treatment modality for a wide range of malignancies. ICIs have been shown to aid in remission of cancers with poor prognosis, including stage IV malignancies. The first ICI was approved for metastatic melanoma, but now ICI indications range from melanoma to small cell lung cancer and triple-negative breast cancer. While this drug class has revolutionized cancer treatment, ICIs can also cause a broad range of immune-related adverse events, including myocarditis. ICI myocarditis has a broad clinical presentation, from fulminant heart failure to cardiac arrhythmia, and is confirmed with a histopathology finding of myocardial infiltration by T lymphocytes and macrophages and cell death. We present a case of cardiogenic shock secondary to ICI-associated myocarditis with onset 13 months after administration of pembrolizumab for triple-negative breast cancer.

**Case Report:**

A 30-year-old female with history of triple-negative breast cancer presented to the emergency department with a 1-month history of progressive chest tightness, dyspnea on exertion, and dry cough. The patient was diagnosed with acute decompensated heart failure (stage C cardiogenic shock), started on milrinone and vasopressin, and transferred to our institution for a higher level of care. At that time, she progressed to stage D cardiogenic shock. A presumptive diagnosis of ICI-associated myocarditis was made within 12 hours, and treatment with high-dose intravenous methylprednisolone was initiated. Despite the high mortality associated with ICI-associated myocarditis, the patient improved and was discharged 9 weeks after admission.

**Conclusion:**

This case highlights the importance of considering ICI myocarditis in the differential diagnosis of cardiogenic symptoms, even in delayed presentations, and supports the lifesaving potential of early recognition and treatment.

## INTRODUCTION

Immunotherapy has revolutionized oncology with the introduction of immune checkpoint inhibitors (ICIs) for cancer treatment. The first ICI approved by the US Food and Drug Administration in 2011 was for metastatic melanoma.^1^ As clinical trials continue to demonstrate the efficacy of ICI use for a broadening range of cancers, the number of approved therapeutic agents grows, with 11 ICIs approved as of July 31, 2023, for 20 different cancer types.^1,2^ Current indications for ICI therapy include refractory melanoma, non–small cell lung cancer, small cell lung cancer, urothelial carcinoma, poor-risk renal cell carcinoma, Merkel cell carcinoma, cutaneous squamous cell carcinoma, hepatocellular carcinoma, and triple-negative breast cancer.^1^ Pembrolizumab, a common ICI, has many indications for use, with clinical trials showing benefit in triple-negative breast cancer.^3-5^ Breast cancer has the highest incidence of malignancies globally, surpassing lung cancer as of 2020, and is the leading cause of cancer mortality in women.^6^ Of the breast cancer subtypes, triple-negative breast cancer has a worse prognosis compared to hormone receptor–positive subtypes.^3^ Although ICI therapy has proven benefit as a cancer therapy, it is associated with many potential immune-related adverse events.^7^

Immune-related adverse events can be caused by autoreactive T cells that damage host tissues and cause a broad range of toxicities, including hepatitis, colitis, myositis, dermatitis, and thyroiditis.^8^ The cardiovascular manifestations of immune-related adverse events include arrhythmias, pericarditis, myocarditis, vasculitis, and Takotsubo-like syndrome.^7,9^ The definitive diagnosis of myocarditis is either by endomyocardial biopsy showing T lymphocytic infiltrates or cardiac magnetic resonance showing myocardial edema with an inflammatory myocardial injury pattern and elevated cardiac troponin.^10^ Additionally, microRNA-analysis and 3D-spatial immunophenotyping are emerging diagnostic tools.^11^ The treatment for immune-related adverse events is a course of high-dose corticosteroids, although reports of other immunosuppressive therapies—including mycophenolate mofetil, tacrolimus, alemtuzumab, and abatacept—have been documented.^10^

We present the case of a patient with ICI myocarditis that presented 13 months after initiation of combination chemotherapy including an ICI.

## CASE REPORT

A 30-year-old female with a history of triple-negative breast cancer diagnosed 13 months prior was treated at an outside institution with paclitaxel, carboplatin, doxorubicin, cyclophosphamide, and pembrolizumab. Six months after completing chemotherapy, she presented to the emergency department with a 1-month history of progressive chest tightness, dyspnea on exertion, and dry cough. Echocardiography upon admission showed a left ventricular ejection fraction (LVEF) of 10% with a nondilated left ventricle. The patient was diagnosed with acute decompensated heart failure (stage C cardiogenic shock) and started on milrinone and vasopressin (doses unavailable).

Despite initial stabilization (mean arterial pressure ≥65 mm Hg, lactate <2.0 mmol/L [reference range, 0.5-2.2 mmol/L], and urine output >0.5 mL/kg/h), her condition deteriorated with respiratory failure requiring bilevel positive airway pressure and increased lactic acidosis 48 hours after admission. Coronary angiography revealed patent coronary arteries. Hemodynamic measurements were left ventricular end diastolic pressure of 19 mm Hg, right atrial pressure of 10 mm Hg, pulmonary artery pressure of 34/24 mm Hg, and pulmonary capillary wedge pressure of 21 mm Hg.

The patient was transferred to our tertiary center for a higher level of care 3 days after presentation, with an intra-aortic balloon pump in place and on milrinone (0.25 μg/kg/min), norepinephrine (0.24 μg/kg/min), and vasopressin (0.04 units/min). On arrival, she was alert and oriented, with a heart rate of 132 beats per minute, respiratory rate of 22 breaths per minute, and oxygen saturation of 99% on room air. She had coarse lung sounds but no respiratory distress. Extremities were slightly cool without edema. Electrocardiogram showed sinus tachycardia (135 beats per minute), low voltage QRS, and nonspecific T-wave abnormalities. Laboratory workup showed troponin I of 103.87 ng/L (reference value, ≤35 ng/L), B-type natriuretic peptide of 4,004 pg/mL (reference range, 0-99 pg/mL), and lactate of 2.0 mmol/L. The patient had progressed to stage D cardiogenic shock.

Advanced therapies were considered. Left ventricular assist device implantation was not feasible because of the patient's small left ventricular cavity, and heart transplant was contraindicated because of her recent cancer. Within 6 hours of arrival, the patient was placed on venoarterial extracorporeal membrane oxygenation (VA-ECMO) as a bridge to recovery. Endomyocardial biopsy was performed peri-cannulation.

A presumptive diagnosis of ICI-associated myocarditis was made within 12 hours, and treatment with high-dose intravenous (IV) methylprednisolone (1 g per day for 3 days) was initiated, followed by a taper. VA-ECMO was converted to central support 48 hours later because of limb ischemia from peripheral cannulation. Abatacept 500 mg IV was administered on hospital days 11 and 20. Endomyocardial biopsy confirmed lymphocytic myocarditis (CD4/CD8 positive, PD-L1 negative, minimal necrosis).

The patient's hemodynamics improved, allowing vasopressor weaning and VA-ECMO decannulation. An Impella 5.5 (Abiomed, Inc) was implanted on hospital day 18 and removed in week 7. Repeat echocardiography at 8 weeks showed LVEF improvement from 10% to 30% to 35%. The patient's hospitalization was complicated by steroid-induced adrenal insufficiency and acute kidney injury that required continuous renal replacement therapy.

She was discharged home 9 weeks postadmission on apixaban 5 mg twice daily and hydrocortisone 15 mg in the morning and 5 mg in the evening, with cardiology, endocrinology, and oncology follow-up.

At a 6-month primary care clinic follow-up, the patient reported a 40-pound weight loss, and 1 month later, she was readmitted after cardiac arrest from which she did not recover.

## DISCUSSION

While the initial randomized controlled trials for ICIs did not monitor prospectively for myocarditis, subsequent retrospective studies estimated the incidence to range from 0.27% to 1.14%.^7^ According to case reports compiled in the World Health Organization pharmacovigilance database and published in 2018, the median time from initiation of ICI therapy to the onset of myocarditis was approximately 30 days.^9^ Our case represents one of few reported incidents of late-onset ICI myocarditis, with symptom onset 13 months after initiation of an ICI. Review of the literature found that the longest period reported between ICI therapy and symptomatic presentation was 2.5 years after the start of pembrolizumab in a patient who experienced an out-of-hospital cardiac arrest attributable to ventricular fibrillation.^12^ Given that the latency period between ICI exposure and clinical presentation can extend beyond 2 years, maintaining a high index of suspicion is essential. Early recognition and prompt initiation of treatment may dramatically reduce mortality, as demonstrated in a case series by Oyakawa et al, where implementation of a troponin T level–based monitoring strategy in patients receiving ICI therapy reduced myocarditis-related mortality from 60% to 0%.^13^

Although our patient received multiple immunosuppressive agents during her breast cancer treatment course, including doxorubicin and cyclophosphamide, several factors point to pembrolizumab as the most likely etiology of her myocarditis. First, the cardiotoxic effects of anthracyclines such as doxorubicin are typically cumulative and dose-dependent and manifest as chronic progressive cardiomyopathy rather than acute fulminant myocarditis.^14^ Additionally, the delayed onset of our patient's symptoms, occurring more than 6 months after the last dose of chemotherapy, makes anthracycline-induced cardiomyopathy less likely. In contrast, pembrolizumab has been directly associated with ICI myocarditis, which is often characterized by acute lymphocytic infiltration, rapid hemodynamic decline, and responsiveness to immunosuppression, all of which were present in this case. Moreover, the absence of alternative causes (such as infection, ischemia, or infiltrative process) supports the diagnosis.

A diagnostic challenge in this case was the endomyocardial biopsy findings. Although the biopsy revealed lymphocytic infiltrates consistent with myocarditis, immunohistochemical staining for PD-L1 was negative. This result was unexpected, as PD-L1 expression on myocardial tissue has been described in prior reports of ICI-associated myocarditis.^15,16^ However, a postmortem analysis of endomyocardial biopsy specimens by Hauck et al demonstrated a substantial sampling error for lymphocytic myocarditis, with a false-negative rate of approximately 37%, underscoring the potential for patchy or focal involvement that evades detection on limited samples.^17^ Given the clinical presentation of new-onset cardiogenic shock in a young patient with prior ICI exposure, the absence of alternative etiologies, the improvement in cardiac function with immunosuppressive therapy, and the histopathologic evidence of lymphocytic infiltration, we considered the diagnosis of ICI-associated myocarditis most likely despite the lack of PD-L1 staining. This case highlights the limitations of endomyocardial biopsy sensitivity in focal myocarditis and reinforces the importance of integrating clinical, histologic, and therapeutic response data when evaluating suspected ICI-related cardiotoxicity.

The rate of mortality or heart transplantation for patients with fulminant myocarditis remains high, with an estimated 10.4% at 30 days and 14.7% at 5-year follow-up.^18^ The mortality rate for ICI-associated myocarditis was 50% in a retrospective pharmacovigilance study.^9^ Our patient was successfully weaned off mechanical support with survival at 6 months postdischarge.

## CONCLUSION

We report a rare case of late-onset ICI-associated myocarditis manifesting with fulminant cardiogenic shock more than a year after initiation of pembrolizumab in a patient with triple-negative breast cancer. Her successful recovery from the initial hospitalization highlights that even for delayed presentations, prompt diagnostic consideration, early immunosuppression, and advanced mechanical support may salvage patients otherwise at high risk. Clinicians prescribing ICIs should maintain a high index of suspicion for myocarditis throughout therapy, and studies are needed to define long-term surveillance strategies and optimal immunomodulatory regimens.

## Notes

*This article meets the Accreditation Council for Graduate Medical Education and the American Board of Medical Specialties Maintenance of Certification competencies for Patient Care and Medical Knowledge.*
